# Trauma und Erinnerung – ein Beitrag zur aktuellen Debatte in Recht und Psychotherapie

**DOI:** 10.1007/s00115-024-01665-x

**Published:** 2024-05-06

**Authors:** Julia Schellong, Anton Schellong, Ursula Gast, Ulrich Frommberger, Alexander Jatzko, Ingo Schäfer

**Affiliations:** 1 Klinik und Poliklinik für Psychotherapie und Psychosomatik, Medizinische Fakultät, Technische Universität Dresden, Fetscherstraße 74, 01307 Dresden, Deutschland; 2 Citywalx GmbH, Wien, Österreich; 3 Praxis für Psychosomatische Medizin, Mittelangeln, Deutschland; 4 Klinik für Psychiatrie und Psychotherapie, Universitätsklinikum Freiburg, Medizinische Fakultät, Albert-Ludwigs-Universität Freiburg, Freiburg, Deutschland; 5 Privatpraxis, Sölden, Deutschland; 6 CuraMed Privatklinik Stillachhaus, Oberstdorf, Deutschland; 7 Klinik für Psychiatrie und Psychotherapie, Universitätsklinikum Hamburg-Eppendorf, Hamburg, Deutschland

**Keywords:** Erinnerung/Fehlbarkeit, Justiz, Erinnerungsprozesse, Sexualisierte Gewalt, Therapeutisches Dilemma, Memory/fallibility, Judiciary, Memory processes, Sexual violence, Therapeutic dilemma

## Abstract

Der Abruf von Erinnerungen an vergangene Ereignisse, Gefühle und Erfahrungen ist ein komplexer Prozess. Wenn wir traumatische Ereignisse erleben, wie es bei sexualisierter Gewalt der Fall ist, ergibt sich eine ganze Reihe zusätzlicher Schwierigkeiten und Komplexitäten. Besonders wichtig wird dies in Gerichtsverfahren, die sich überwiegend oder ausschließlich auf die Aussage des Opfers stützen, wo das Problem der Fehlbarkeit des Gedächtnisses in den Mittelpunkt rückt. Einige Forschungsarbeiten betonen die Möglichkeit, Erinnerungen hervorzurufen, zu verändern oder zu unterdrücken, insbesondere im Rahmen einer Psychotherapie. Dies hat zu der bedauerlichen Tatsache geführt, dass die Aussagen von Betroffenen, die sich einer Psychotherapie unterzogen haben, häufig als unzuverlässig angesehen werden. Dies wiederum kann zu dem Eindruck führen, dass eine Entscheidung zwischen der Behandlung der negativen Auswirkungen traumatischer Ereignisse und der Maximierung der Chancen für eine Verurteilung des Täters vor Gericht getroffen werden müsse. Der vorliegende Beitrag führt in einige zentrale Konzepte unseres derzeitigen Verständnisses von Erinnerung ein und gibt einen Überblick über die einschlägige wissenschaftliche Literatur und Debatte. Anschließend wird das Dilemma in Bezug auf die verschiedenen Gruppen aller Beteiligten (das heißt Betroffene, Justizangehörige und Psychotherapeut:innen) untersucht. Schließlich wird ein Rahmen für die Lösung dieses Problems vorgeschlagen, wobei der Schwerpunkt auf der Forschung in entscheidenden Bereichen, auf der Erweiterung von Therapierichtlinien und Dokumentationsverfahren sowie auf der Kommunikation dieser Bemühungen an alle Beteiligten liegt.

## Abstract (en)

The recall of memories of past events, experiences and emotions is a complex process. When experiencing traumatic events, as is the case with sexual violence, a host of additional complexities and difficulties arise. This becomes especially important in court cases which rely mostly or exclusively on the testimony of the victim, where the problem of the fallibility of memory takes center stage. Some research studies emphasize the possibility of inducing, altering or suppressing memories, especially in the context of psychotherapy. This has led to the unfortunate reality that the testimony of victims who have undergone psychotherapy is often considered to be unreliable. This in turn can lead to the impression that a decision has to be made between treatment of the adverse effects of traumatic events and maximizing the chances for a conviction of the perpetrator in court. This article introduces some central concepts of our current understanding of memory and gives an overview of the relevant scientific literature and debate. Following this, it examines the dilemma as it pertains to the different groups of all involved parties (i.e., victims, members of the judiciary and psychotherapists). Lastly, it proposes a framework of how to approach a solution to this problem by focusing on research in critical areas, expansion of therapy guidelines and documentation procedures as well as communication of these efforts to all parties involved.

Das Spannungsverhältnis zwischen der Realität, dass sexualisierte Gewalt häufig vorkommt, und der Möglichkeit, verfälschte Erinnerungen zu induzieren, stellt alle Beteiligten vor komplexe Herausforderungen. Insbesondere im Kontext der juristischen Aufklärung von Straftaten gegen die sexuelle Selbstbestimmung wogt seit Jahren eine Debatte, dass Psychotherapie die Glaubhaftigkeit von Aussagen im juristischen Kontext beschädigen könnte. Eine Entscheidung zwischen Gerechtigkeit und Gesundheit sollte in einem modernen Sozial- und Rechtssystem keinem der Beteiligten aufgebürdet werden.

## Aspekte des wissenschaftlichen Verständnisses von Erinnerungsprozessen

Erinnerung wird als „spezielles, ins Bewusstsein tretendes Ereignis bzw. Erlebnis“ beschrieben [16]. Das autobiografische episodische Gedächtnis spielt eine Schlüsselrolle bei der Speicherung vergangener persönlicher Erinnerungen sowie bei der Identitätsbildung. Es hilft uns im Umgang mit negativen Emotionen und Erfahrungen, aber auch dabei, ein soziales Netzwerk aufzubauen und aufrechtzuerhalten, ein kontinuierliches Selbstgefühl zu entwickeln und unser zukünftiges Verhalten zu steuern. Diese vier Funktionen stehen in Zusammenhang mit psychischem und körperlichem Wohlbefinden [45]. Die komplexe Natur von Erinnerung kann allerdings im juristischen Zusammenhang zu Problemen und Dilemmata führen [11, 36].

Die Kognitionsforschung geht davon aus, dass wir ein dreidimensionales Modell unserer Umwelt kreieren, das über die Sinne ein fortlaufendes Update erhält. Dabei spielt Plastizität in neuronalen Netzwerken eine zentrale Rolle [4]. Eine Gedächtnisspur zieht sich quer durch das Gehirn, sodass viele Regionen hieran beteiligt sind, beispielsweise die Sehzentren, limbische, sensorische oder motorische Areale. Orchestriert wird dies vom Hippocampus [18]. Insgesamt zeigt sich ein empfindliches Wechselspiel zwischen Erregung und Hemmung, das heißt, wie Reize im Gedächtnis wahrgenommen, gespeichert, abgerufen und verstärkt oder abgeschwächt werden. Erinnerungen, positive wie negative, sind ein zentrales Instrument, um uns durch das Leben zu führen. Negative Erinnerungen sind leichter abrufbar, werden verzögert vergessen, lebendiger erinnert, dafür allerdings weniger überprüft und nur selektiert erinnert [49].

Erinnerung muss als differenzierter Prozess verstanden werden. Aktuelle Konzepte empfehlen, sich dies zunutze zu machen, und plädieren dafür zu trainieren, bestimmte, quälende Formen der Erinnerungen an ein aversiv erlebtes Ereignis gezielt zu unterdrücken, wenn diese zu einer erheblichen Beeinträchtigung der Lebensqualität führen [34]. Ein anderer Forschungsstrang stellt die Frage nach der individuellen selbstrelevanten emotionalen Valenz von traumatisch erlebten Erinnerungen [44] und diskutiert Besonderheiten traumatischer Erinnerungsfragmente im Unterschied zur Erinnerung an nichttraumatische traurige Erlebnisse [31]. Besonders im Zusammenhang mit stark mit Erinnerung verbundenen Krankheitsbildern wie Depressionen und posttraumatischer Belastungsstörung (PTBS) werden Einbußen in der Spezifität (das heißt Erinnerungen an ein bestimmtes, zeitlich zugeordnetes Erlebnis) und der Kohärenz (narrativer Ausdruck der Gesamtstruktur der autobiografischen Erinnerungen) des Gedächtnisses festgestellt [33, 45]. Auffälligkeiten finden sich zudem bei dissoziativen Störungen inklusive der dissoziativen Identitätsstörung, bei denen eine veränderte Gedächtnisfunktion den wesentlichen psychopathologischen Kern darstellt [47].

Erinnerungsprozesse sind in unterschiedliche funktionale Bereiche aufgegliedert

Die Forschung zeigt, dass Erinnerungsprozesse in unterschiedliche funktionale Bereiche aufgegliedert und abhängig von den jeweiligen Umständen sind [34, 49]. Jede Theorie von Erinnerung unterscheidet vielfältige Formen, die zwar miteinander vernetzt sind, aber trotzdem erhebliche Unterschiede in ihrer Funktionsweise und Auswirkung aufweisen. Als Beispiel sei die Unterscheidung in das deklarative unwillkürliche Gedächtnissystem, das nichtdeklarative unwillkürliche Gedächtnissystem und das deklarative willkürliche Gedächtnissystem ausgeführt [20, 46]. Dieses Modell der multiplen Erinnerungssysteme ist dem Verständnis des Kontexts von sexualisierter Gewalt und Trauma besonders förderlich.

Gemäß diesem Modell ist das *deklarative unwillkürliche* Gedächtnissystem durch unerwünschte, emotional aversive Erinnerungen gekennzeichnet, die unaufgefordert in Form von sensorischen Bildern in den Sinn kommen. Dies ähnelt einem Kernsymptom der PTBS – dem unwillkürlichen Wiedererleben des traumatischen Ereignisses in Form von sich aufdrängenden Erinnerungen und Flashbacks [8].

Das *nichtdeklarative unwillkürliche* Gedächtnissystem ist verbunden mit automatischen psychophysiologischen Reaktionen, wobei diese Reaktionen typischerweise durch traumabezogene Hinweise ausgelöst werden, aber auch spontan auftreten können. Hierin spiegeln sich charakteristische PTBS-Symptome wie erhöhte physiologische Reaktionen auf traumabezogene Reize, Hyperarousal und Hypervigilanz wider.

Das *deklarative willkürliche* Gedächtnissystem umfasst hingegen absichtlich erinnerte Episoden und Fakten, die geäußert werden, wenn man sich bewusst entscheidet, über das Trauma zu berichten. Der Bericht eines stattgefundenen Ereignisses ist für die diagnostische Einordnung relevant, aber auch für die Anzeigeerstattung eines strafrechtlich relevanten Ereignisses.

Dieses Modell verdeutlicht die klinische Beobachtung, dass bestimmte, in diesem Falle die unwillkürlichen Modi der Erinnerung Leiden verursachen und daher Ziel leitliniengerechter traumafokussierter psychotherapeutischer Behandlung sind, während andere, die willkürlichen, für juristische Aussagen bedeutsam sind und durch eine Behandlung auch gestärkt werden können [14, 20, 22].

## Wissenschaftliche und juristische Kontroversen um veränderte und beeinflusste Erinnerungen

Dass Erinnerungen beeinflussbar sind, zeigte eine Vielzahl von Studien [9, 30, 37]. Äußere Einflüsse, beispielsweise suggestive Befragungen, können das Gedächtnis verändern [5, 30]. Scheinerinnerungen, die keine Erlebnisgrundlage haben, können subjektiv für wahr gehalten werden und sich dann phänomenologisch nicht von echten Erinnerungen unterscheiden [11]. Interessanterweise scheint es weit mehr Studien zu Scheinerinnerungen zu geben als Studien über das Suggerieren davon, dass Erlebnisse nicht geschehen sind, wobei Letzteres zu Auslassungsfehlern bzw. falschen Nichterinnerungen führen kann. Selbst innere Einflüsse wie Lügen und Verschweigen können zu Veränderungen in der Überzeugung führen, ob ein Ereignis stattgefunden hat [7, 28, 30]. Menschen kann suggeriert werden, dass Ereignisse oder Details davon vorhanden waren, obwohl dies in Wirklichkeit nicht zutraf [6]. Es kann aber auch fälschlicherweise suggeriert werden, dass bestimmte Ereignisse oder Details nicht erlebt wurden, obwohl sie tatsächlich vorhanden waren [13, 38]. Auch dies sei bei Vernehmungen zu beachten [30].

Erst das Aussprechen von Erinnerungen macht diese juristisch relevant

Darüber hinaus bedeutet das Versäumnis, über ein traumatisches Ereignis wie etwa sexuellen Missbrauch zu berichten, nicht, dass man sich überhaupt nicht daran erinnern kann [26]. Oft werde eine aversive Erinnerung „subjektiv“ vergessen, das heißt unterdrückt und sei dann nicht mehr präsent, hätte bei Nachfrage jedoch hervorgeholt werden können. Dieses subjektive Vergessen steht in positivem Zusammenhang mit stärkerer Traumasymptomatik im Erwachsenenalter und dem Verschweigen von Missbrauch in der Kindheit [51]. Eine psychotherapeutische Behandlung kann dann die Bedingungen bieten, solche Erinnerungen auszusprechen [2, 3]. Erst das Aussprechen von Erinnerungen macht diese juristisch relevant.

## Erläuterung der Dilemmata der beteiligten Gruppen

Rechtsprechung und Gesellschaft stehen vor der Herausforderung, faire Verfahren und Unterstützung für Opfer zu gewährleisten, ohne dabei die Rechte der Beschuldigten zu vernachlässigen. Insgesamt bleibt die Thematik komplex und erfordert eine ausgewogene Betrachtung der psychotherapeutischen, rechtlichen und ethischen Aspekte. Es sind hierbei ein Interessenkonflikt und eine Rechtsgüterabwägung wissenschaftlich und ethisch auszuloten, die interdisziplinär bislang ungelöst sind: Wie kann der Schutz von Opfern gewährleistet und gleichzeitig sichergestellt werden, dass die Rechte aller Beteiligten, insbesondere auch möglicher Beschuldigter, respektiert werden?

### Dilemma der Betroffenen

Weltweit hat etwa ein Drittel aller Frauen einmal im Leben sexualisierte Gewalt erlebt, wobei die Prävalenzschätzungen sehr unterschiedlich sind [17]. Das Bundeskriminalamt (BKA) betont, dass Straftaten gegen die sexuelle Selbstbestimmung in Deutschland über alle gesellschaftlichen Gruppen hinweg ein Problem darstellen, mit einem hohen Anteil an Fällen im sozialen Nahfeld. Die Dunkelziffer liege dabei deutlich höher als die rund 118.000 gemeldeten Fälle im Jahr 2022 [12].

Langzeitfolgen können sich unter anderem im Beziehungsalltag, in der gesundheitlichen Verfassung, im Beruf und in der sozialen Einbindung der Betroffenen zeigen [24, 35]. In einigen Fällen werden psychische Folgeerscheinungen wie eine PTBS erst nach Jahren aktiv [40].

Opfern sexueller Straftaten stehen vielfältige Rechte zu und Unterstützungsmöglichkeiten beiseite [50]. Zudem besteht nach dem im Sozialgesetzbuch (SGB) XIV geregelten Sozialen Entschädigungsrecht (SER) Anspruch auf ein Fallmanagement über die Versorgungsbehörde bei psychischen Beschwerden, auf schnelle Hilfe in einer Traumaambulanz (siehe Giesmann et al. in dieser Ausgabe, https://projekt-hilft.de) sowie auf ausreichende gesundheitliche Versorgung und Psychotherapie [41].

Im günstigsten Fall hat die Opferzeugenvernehmung nach einer Straftat schon vor einer Psychotherapie stattgefunden. Ein Dilemma zwischen Gesundheit und Gerechtigkeit kann dennoch auftreten, wenn sich Symptome einer PTBS entwickelt haben und die Empfehlung zu einer traumafokussierten Behandlung zeitgleich mit einem laufenden Prozess im Raum steht. Wichtig ist zu betonen, dass traumafokussierte Verfahren deutlich höhere Effektstärken zeigen als nichttraumafokussierte [35].

Nicht immer gelingt es Betroffenen, sich zeitnah an Strafverfolgungsbehörden zu wenden

Nicht immer gelingt es Betroffenen, sich zeitnah an Strafverfolgungsbehörden zu wenden. Häufig warten sie viele Jahre, bevor sie etwas preisgeben, und die Offenlegung erfolgt in der Regel stückweise, wenn das Vertrauen zum Empfänger aufgebaut ist [2, 3]. Häufige Gründe für eine verlängerte Offenbarungslatenz waren ein jüngeres Alter zu Beginn des Missbrauchs, innerfamiliäre Beziehungen zum Täter [21] sowie Scham und Schuldgefühle [1]. Nicht selten sind Psychotherapeut:innen die ersten, denen sich Betroffene offenbaren.

Doch der Verdacht der Beeinflussung der Erinnerung durch Psychotherapie verschlechtert die Chancen von Behandelten, dass ihren Angaben Glauben geschenkt wird, und zwar maßgeblich auch weil der Bundesgerichtshof (BGH) dort eine „besondere Glaubhaftigkeitsprüfung“ fordert (4 StR 1/17 – 22.06.2017; [11]). Eine zusätzliche Schwierigkeit entsteht, wenn das Erinnern an ein traumatisches Erlebnis im Rahmen des psychotherapeutischen Prozesses erstmalig stattgefunden hat und der Wunsch nach Anzeige entsteht.

Im Ergebnis entsteht für Betroffene eine unhaltbare Situation. Einerseits haben sie ein Recht auf eine wirksame psychotherapeutische Behandlung der Folgen von erlittener sexualisierter Gewalt, andererseits haben sie einen Anspruch darauf, den Staat anzuhalten, diese Verbrechen strafrechtlich zu verfolgen. Dass sich diese beiden Rechte in ihrer Geltendmachung gegenseitig im Wege stehen, ist mit einer modernen Rechtsordnung unvereinbar, vor allem dann, wenn dieser Widerspruch von den Betroffenen selbst getragen werden muss.

### Dilemma der Justizangehörigen

Der Blick auf Erinnerung in Zeugenaussagen aus juristischer Perspektive folgt dem definierten Ziel der Tatsachenfeststellung und der Beweiswürdigung [42]. Bei der Konstellation Aussage gegen Aussage, wie sie bei sexualisierter Gewalt häufig ist, sind Richter in den Urteilsbegründungen angehalten, Aspekte der Empfänglichkeit für Suggestion, aktives Suggerieren, die Plausibilität des Ereignisses und des zwischenzeitlichen Nichterinnerns, die Generierung bildhafter Vorstellungen und Quellenverwechslungsfehler zu berücksichtigen [48]. Die sogenannten Wormser Prozesse Ende der 1990er-Jahre [42] waren diesbezüglich ein großer Einschnitt in der Beurteilungsgenese. Für eine Verurteilung aufgrund einer einzigen belastenden Aussage bestehen seither verbindliche methodische Vorgaben [11], wie das hypothesengeleitete Vorgehen in Glaubhaftigkeitsbeurteilungen, die methodische Annahme der Unwahrheit der Aussage („Nullhypothese“) bzw. der Gedächtnisverfälschung („Suggestionshypothese“) sowie die Überprüfung dieser Hypothesen durch Falsifizierung [11]. Als hoch im Verdacht stehender Umstand gilt, dass die Erinnerung stark beeinflusst worden sein könne, wenn Opferzeugen den Vorfall ausführlich mit Therapeut:innen, Hilfeeinrichtungen oder Familienangehörigen besprochen haben, was zur Unwiderlegbarkeit der „Suggestionshypothese“ führe [11]. Insbesondere das Erinnern eines traumatischen Ereignisses nach langer Zeit wird verdächtigt, therapeutisch suggeriert worden zu sein [37], und dies umso mehr, wenn es erstmals innerhalb der Psychotherapie stattfindet [14].

Eine Vielzahl wissenschaftlicher Veröffentlichungen diskutiert die Möglichkeit, falsche oder verfälschte Erinnerungen zu erzeugen [9, 37]. Allerdings wird auch immer häufiger betont, dass das Suggerieren von Nichterfahrungen gegenüber Opfern, Zeugen und Verdächtigen weniger beachtet würde. Es könne beispielsweise genauso zu Fehlidentifikationen und falschen Geständnissen führen, wenn die Täter ihre Opfer zum Schweigen brächten und behaupteten, es sei nichts geschehen [30]. Andere neue Befunde sind, dass Fälle von wiedererlangten Erinnerungen in einem therapeutischen Umfeld nur eine Minderheit der Fälle von wiedererlangten Erinnerungen an traumatische Ereignisse darstellen und dass dies unabhängig von der Art der Therapie zu sein scheint [15].

Es ist gut belegt, dass traumafokussierte Therapien die wirksamsten Ansätze zur Behandlung der PTBS darstellen, was seinen Niederschlag in entsprechenden evidenzbasierten Empfehlungen gefunden hat [35]. Dem steht jedoch entgegen, dass genau diese Therapietechniken besonders häufig in den Verdacht geraten, Erinnerungen zum Schaden der Glaubwürdigkeit zu verändern [23, 29].

Das Dilemma der Justizbeamt:innen liegt also im aus historisch guten Gründen erwachsenen Misstrauen gegenüber im beraterischen oder psychotherapeutischen Kontext berichteten oder aufgetretenen Erinnerungen und im verlorenen Vertrauen, dass der therapeutische Umgang mit Erinnerungen so erfolgt, dass die Glaubhaftigkeit nicht beschädigt wird. Dadurch kann der Eindruck entstehen, dass zwischen Psychotherapie und Glaubhaftigkeit entschieden werden müsse.

### Dilemma der Psychotherapeut:innen

Ziel psychotherapeutischer Behandlung ist das Lindern von Leid und die Erarbeitung von Perspektiven für die Zukunft und nicht das Bemühen, Erinnerung wiederzufinden [9]. Allerdings stehen am Anfang jeder medizinischen Behandlung parallel zum Aufbau einer tragfähigen therapeutischen Beziehung eine Anamneseerhebung und eine differenzierte Diagnostik. Für beides ist es unabdingbar, auch nach Belastungen und potenziell traumatischen Erlebnissen im bisherigen Leben zu fragen und deren Bedeutung für die aktuelle psychische Befindlichkeit einzuschätzen. Eine verantwortliche Psychotherapeut:in ist sich dabei immer bewusst, dass nicht nach solchen Erinnerungen „gesucht“ werden soll, aber gleichzeitig ein Rahmen geboten werden soll, um das Mitteilen entsprechender Erinnerungen zu ermöglichen [9, 36]. Sobald von einer Straftat berichtet wird, stehen Therapeut:innen allerdings vor der Herausforderung, die Bedeutung von Psychotherapie bezüglich juristischer Belange mit zu bedenken und darüber aufzuklären [11].

Kognitionswissenschaftliche Erkenntnisse haben in dieser Konstellation einen neuen Stellenwert. Sie sind wichtig, um für innovative Therapieverfahren offen zu sein. Darüber hinaus schärfen sie das Bewusstsein, dass jede menschliche Kommunikation und insbesondere diejenige mit einem hohen Vertrauensbonus, wie in der therapeutischen Bindung, besonderen Einfluss auf das Erinnerungssystem birgt. Spezialisierte Traumatherapie kann helfen, mit den Auswirkungen sexualisierter Gewalt umzugehen. Bei traumafokussierten Ansätzen steht im Zentrum die Linderung von Symptomen, bei denen davon ausgegangen wird, dass sie auf das deklarative und nichtdeklarative *un*willkürliche Gedächtnis zurückzuführen sind, wie Intrusionen und psychophysiologischen Beschwerden [20]. Zwar bedarf es hierzu eines Wachrufens von Erinnerungsspuren [25], es ist aber nie das Ziel, den Erinnerungsinhalt an sich zu verändern. Das willkürliche Gedächtnis lässt sich durch psychotherapeutische Unterstützung sogar stärken [14, 22], es kann allerdings auch empfänglicher für spontane Fehlerinnerungen werden [23].

Entsprechend hält die Kontroverse um die Anwendung traumafokussierter Verfahren während eines laufenden Gerichtsverfahrens weiter an [11, 36]. So fand eine Untersuchung zu Augenbewegungen, wie sie bei „eye movement desensitization and reprocessing“ (EMDR) eingesetzt werden, zwar mehr korrekte Erinnerungen, aber eine Erhöhung spontaner Fehlerinnerungen [23]. Insbesondere Verfahren, die mit Bildern arbeiten, wie imaginative Verfahren, stehen im Verdacht, Erinnerungsphänomene zu verfälschen oder gar Scheinerinnerungen zu produzieren [27]. Jüngere Untersuchungen [20] stellen dies infrage; sie haben ergeben, dass sich die Anzahl der richtigen Details im freien Abruf bei der Gruppe, die das imaginative Verfahren erhalten hatte, sogar verbesserte, nicht aber bei der Kontrollgruppe. Diese Ergebnisse aus der Laborforschung stehen im Einklang mit der Beobachtung, dass während einer Strafverfolgung wegen sexuellen Kindesmissbrauchs die Erinnerungsbeständigkeit 10–16 Jahre später und die Inanspruchnahme einer Therapie positiv korrelierten [22].

Trotz dieser ermutigenden Ergebnisse bleibt in der Arbeit mit psychischen Folgen strafrechtlich relevanter Erinnerungen das Dilemma, durch psychotherapeutisches Arbeiten einerseits ein vertrauensvolles therapeutisches Bündnis zu etablieren, in dem mit großer Offenheit traumatische Erlebnisse berichtet werden können, andererseits aber in die Gefahr zu geraten, die Glaubhaftigkeit von Aussagen der Behandelten im juristischen Prozess zu beschädigen.

## Schlussfolgerungen für die psychotraumatologische Arbeit

Aus den oben ausgeführten Dilemmata lassen sich einige Implikationen für den Umgang mit Erinnerungen während eines laufenden oder angestrebten Gerichtsverfahrens ableiten. Die Psychotraumatologie sollte dies als Ansporn sehen, mithilfe von Forschung, Leitlinien, guten Dokumentationsanleitungen und Kommunikation das Vertrauen Justizangehöriger darin zu stärken, dass eine psychotraumatologische Behandlung auch dazu beitragen kann, die Verfälschung von strafrechtlich relevanten Erinnerungen zu minimieren.

Eine unterstützende Umgebung und Validation sind für die Heilung von Menschen, die von sexualisierter Gewalt betroffen sind, entscheidend. Traumainformierte Gesprächsführung sollte allen Berufsgruppen selbstverständlich sein, die mit von sexualisierter Gewalt Betroffenen arbeiten [39]. Erinnerungen, die erst später im Leben oder im Zuge einer Therapie wiederkehren oder neu auftreten, können wahr, falsch oder eine Mischung aus beidem sein [9]. Sie bedürfen eines erhöhten Grades an Vorsicht im Umgang, vor allem im Zusammenhang mit Vorgängen der Justiz. So ist es etwa von therapeutischer Seite unbedingt zu vermeiden, aus Verhaltensmerkmalen auf das Vorhandensein „verschütteter“ Erinnerungen zu schließen und zu forcieren, solche „freizulegen“ [9, 14]. Eine reflektierte und sensible Herangehensweise ermöglicht es Therapeut:innen, effektiv mit Menschen zu arbeiten, deren Gedächtnisprozesse möglicherweise beeinflusst oder umstritten sind, und gleichzeitig die Integrität der therapeutischen Beziehung zu bewahren.

Therapeutische Interventionen, die auf psychische Stabilisierung in der Gegenwart fokussieren, gelten als risikoarm in Bezug darauf, Einfluss auf Erinnerungsprozesse zu nehmen [36]. Sie genügen aber bei Vorliegen einer PTBS nicht der erforderlichen Behandlung. Die leitliniengerechte traumafokussierte Therapie konzentriert sich hauptsächlich auf traumatische Erinnerungen bzw. Intrusionen, die als unkontrollierte unwillkürliche Erinnerungen wiederkehren [8], und zielt darauf ab, deren belastende und intrusive Auswirkungen zu verringern [9, 35]. Unerwünschte Erinnerungen erfordern allerdings die reflexive Ausrichtung der Aufmerksamkeit und den Zugang zum Bewusstsein, um beispielsweise durch hemmende Kontrolle wirksam unterdrückt zu werden [25]. Behandlungen, die dies berücksichtigen, wie traumafokussierte Verfahren, sollten das realitätsgerechte willkürliche Abrufen von strafrechtlich relevanten Erinnerungen nicht beeinträchtigen, sondern wenn möglich sogar stärken.

Gefragt ist weitere Forschung zu Erinnerungsveränderungen in ihrer Vielfältigkeit, unter anderem mit den Mitteln der Laborforschung in einer virtuellen Umgebung [43], im klinischen Kontext und im Kontext von Aussagesituationen [19]. Beispiele sind Forschungsarbeiten zu einzelnen in Psychotherapie und traumafokussierter Therapie [25] adressierten Symptomen und zu spezifischen in der Therapie angewendeten Techniken [20, 23]. Wichtig sind aber auch klinische Studien, die zusätzlich kognitionswissenschaftliche Aspekte in Bezug auf rechtswissenschaftliche Fragen berücksichtigen [19], genauso wie Forschung bezüglich der vielfältigen Einflüsse auf Zeugenaussagen bei Anzeigen von sexualisierter Gewalt oder Aussagen vor Gericht auch außerhalb einer Psychotherapie [11, 32]. Diese Fragen und Ergebnisse sollten sich in der Überarbeitung klinischer Leitlinien zu allen traumaassoziierten psychischen Störungen niederschlagen und dadurch in der Praxis verankern. Ihre Überprüfung könnte zudem in einer etwaigen Begutachtung bei strittigen Fällen herangezogen werden.

Ein wesentliches Element ist sicherlich die differenzierte Dokumentation

Ein wesentliches Element ist sicherlich die differenzierte Dokumentation, insbesondere wenn tatrelevante Informationen thematisiert werden, inklusive der inhaltlichen Ausgestaltung der Aussage bzw. der Befragungsmodalitäten, die diese hervorgerufen haben [11, 14, 36]. Wünschenswert sind die Erarbeitung, Erprobung und Akkreditierung von Dokumentationstools, die vor dem Einsatz traumafokussierter Techniken eingesetzt werden und auch für Justizangehörige und Begutachtende transparent gemacht werden können, sodass sich diese auf eine korrekte Anwendung der Leitlinien verlassen können [10, 11].

Ein weiteres wesentliches Element ist die Kommunikation mit allen Beteiligten. Therapeut:innen sind nach dem Patientenrechtegesetz verpflichtet, über Psychotherapie und das vorgesehene spezielle Verfahren aufzuklären. Eine transparente Kommunikation über ihre therapeutischen Ansätze sollte unter anderem die möglichen Auswirkungen auf Erinnerungen und Erinnerungsverfälschungen beinhalten. Ein Bewusstsein für ethische Grundsätze ist von entscheidender Bedeutung, um sicherzustellen, dass die Therapie die Autonomie und das Wohlbefinden der Behandelten gleichermaßen respektiert. Darüber hinaus sollte die Kommunikation fachübergreifend stattfinden, und die besonderen Belange von Menschen, die sexualisierte Gewalt erlebt haben, sollten sich in Schulungen aller Berufsgruppen niederschlagen.

## Fazit für die Praxis

- Die Wechselwirkungen zwischen Kognitionswissenschaft und Folgen sexualisierter Gewalt sind komplex. Die differenzierte Natur von Erinnerungen, die Kontextabhängigkeit beim Abruf der Erinnerungen und deren Beeinflussbarkeit umfassend zu verstehen, ist entscheidend, um gerechte Verfahren, angemessene Unterstützung für Opfer und eine ausgewogene Berücksichtigung der Rechte aller Beteiligten sicherstellen zu können.
- Therapeut:innen, Jurist:innen und die Gesellschaft insgesamt stehen vor der Herausforderung, ethische und wissenschaftlich fundierte Ansätze zu entwickeln, um eine Therapie möglich zu machen, ohne dass diese im Generalverdacht steht, prozessrelevante Erinnerungen unglaubwürdig zu machen.
- Insgesamt erfordert das parallele Bewusstsein für die Möglichkeit von Erinnerungsbeeinflussungen einerseits und den Behandlungsbedarf angesichts psychischer Folgen sexualisierter Gewalt andererseits ein ausgewogenes und einfühlsames Herangehen an die therapeutische Praxis sowie eine kontinuierliche Reflexion über ethische Standards und Forschungsergebnisse.
- Ziel ist ein multidisziplinärer Ansatz, der die Zusammenarbeit aller beteiligten Gruppen, einschließlich der Betroffenen, fördert und die jeweiligen Rechte aller im Blick hat.

## References

[1] Aakvaag HF, Thoresen S, Wentzel-Larsen T, Dyb G, Røysamb E, Olff M (2016). Broken and guilty since it happened: A population study of trauma-related shame and guilt after violence and sexual abuse. J Affect Disord.

[2] Alaggia R, Collin-Vézina D, Lateef R (2019). Facilitators and Barriers to Child Sexual Abuse (CSA) Disclosures: A Research Update (2000–2016). Trauma Violence Abuse.

[3] Alyce S, Taggart D, Sweeney A (2023). Centring the voices of survivors of child sexual abuse in research: an act of hermeneutic justice. Front Psychol.

[4] Anacker C, Hen R (2017). Adult hippocampal neurogenesis and cognitive flexibility—linking memory and mood. Nat Rev Neurosci.

[5] Arce R, Selaya A, Sanmarco J, Fariña F (2023). Implanting rich autobiographical false memories: Meta-analysis for forensic practice and judicial judgment making. Int J Clin Health Psychol.

[6] Azad T, Lindsay DS, Zaragoza MS (2022). Can suggestions of non-occurrence lead to claims that witnessed events did not happen?. J Gen Psychol.

[7] Battista F, Otgaar H (2022). Research on the Effects of Lying on Memory: A Scientometric Analysis and a Call for New Studies. Front Psychol.

[8] Berntsen D (2021). Involuntary autobiographical memories and their relation to other forms of spontaneous thoughts. Philos Trans R Soc Lond B Biol Sci.

[9] Brewin CR, Andrews B (2017). Creating Memories for False Autobiographical Events in Childhood: A Systematic Review. Appl Cogn Psychol.

[10] Bublitz C (2020). Gesundheit oder Glaubhaftigkeit? Auswege aus dem traumatherapeutischen Dilemma. Ethik Medizin.

[11] Bublitz JC (2023) When is Disbelief Epistemic Injustice? Criminal Procedure, Recovered Memories, and Deformations of the Epistemic Subject. Crim Law Philos: 1–28

[12] Bundeskriminalamt (2023).

[13] Clark A, Nash RA, Fincham G, Mazzoni G (2012). Creating non-believed memories for recent autobiographical events. Plos One.

[14] Dodier O, Barzykowski K, Souchay C (2023). Recovered memories of trauma as a special (or not so special) form of involuntary autobiographical memories. Front Psychol.

[15] Dodier O, Patihis L, Payoux M (2019). Reports of recovered memories of childhood abuse in therapy in France. Memory.

[16] Dorsch. (2016). „Erinnerung im Dorsch Lexikon der Psychologie.“ Retrieved 03.01.2024, from https://dorsch.hogrefe.com/stichwort/erinnerung.

[17] Dworkin ER, Krahé B, Zinzow H (2021). The Global Prevalence of Sexual Assault: A Systematic Review of International Research Since 2010. Psychol Violence.

[18] Ekstrom AD, Ranganath C (2018). Space, time, and episodic memory: The hippocampus is all over the cognitive map. Hippocampus.

[19] Fedele E, Trousset V, Schalk T, Oliero J, Fovet T, Lefevre T (2023). Identification of Psycho-Socio-Judicial Trajectories and Factors Associated With Posttraumatic Stress Disorder in People Over 15 Years of Age Who Recently Reported Sexual Assault to a Forensic Medical Center: Protocol for a Multicentric Prospective Study Using Mixed Methods and Artificial Intelligence. JMIR Res Protoc.

[20] Ganslmeier M, Ehring T, Wolkenstein L (2023). Effects of imagery rescripting and imaginal exposure on voluntary memory. Behav Res Ther.

[21] Gewehr E, Hensel B, Volbert R (2021). Predicting disclosure latency in substantiated cases of child sexual abuse. Child Abuse Negl.

[22] Goodman G, Goldfarb D, Quas J, Lyon A (2017). Psychological Counseling and Accuracy of Memory for Child Sexual Abuse. Am Psychol.

[23] Houben STL, Otgaar H, Roelofs J, Smeets T, Merckelbach H (2020). Increases of correct memories and spontaneous false memories due to eye movements when memories are retrieved after a time delay. Behav Res Ther.

[24] Klinger-König, J., K. Berger and H. J. Grabe (2023). “Childhood Trauma and Somatic and Mental Illness in Adulthood—Findings of the NAKO Health Study.” Dtsch Arztebl Int(Forthcoming). 10.3238/arztebl.m2023.0225 PMC10916765 37876295

[25] Legrand N, Etard O, Viader F, Clochon P, Doidy F, Eustache F, Gagnepain P (2022). Attentional capture mediates the emergence and suppression of intrusive memories. iScience.

[26] McNally RJ, Geraerts E (2009). A New Solution to the Recovered Memory Debate. Perspect Psychol Sci.

[27] Muschalla B, Schönborn F (2021). Induction of false beliefs and false memories in laboratory studies—A systematic review. Clin Psychol Psychother.

[28] Otgaar H, Baker A (2018). When lying changes memory for the truth. Memory.

[29] Otgaar, H., S. T. L. Houben, E. Rassin and H. Merckelbach (2021). “Memory and eye movement desensitization and reprocessing therapy: a potentially risky combination in the courtroom.” Memory 29(9): 1254–1262. 10.1080/09658211.2021.1966043 34404311

[30] Otgaar H, Mangiulli I, Battista F, Howe ML (2023). External and internal influences yield similar memory effects: the role of deception and suggestion. Front Psychol.

[31] Perl O, Duek O, Kulkarni KR, Gordon C, Krystal JH, Levy I, Harpaz-Rotem I, Schiller D (2023). Neural patterns differentiate traumatic from sad autobiographical memories in PTSD. Nat Neurosci.

[32] Persson, S. and K. Dhingra (2022). “Moderating Factors in Culpability Ratings and Rape Proclivity in Stranger and Acquaintance Rape: Validation of Rape Vignettes in a Community Sample.” J Interpers Violence 37(13–14): Np11358–np11385. 10.1177/0886260521991294 PMC9253925 33554731

[33] Petzold M, Bunzeck N (2022). Impaired episodic memory in PTSD patients—A meta-analysis of 47 studies. Front Psychiatry.

[34] Phelps EA, Hofmann SG (2019). Memory editing from science fiction to clinical practice. Nature.

[35] Schäfer I (2020). Diagnostik und Behandlung der posttraumatischen Belastungsstörung. Psychotherapeut.

[36] Schemmel J, Volbert R (2021). Therapie oder Glaubhaftigkeit? – Psychotherapeutische Behandlung bei laufenden Strafverfahren [Therapy or Credibility?—Psychotherapy during ongoing criminal proceedings.

[37] Scoboria A, Wade KA, Lindsay DS, Azad T, Strange D, Ost J, Hyman IE (2017). A mega-analysis of memory reports from eight peer-reviewed false memory implantation studies. Memory.

[38] Shepp V, O’Callaghan E, Ullman SE (2020). Interactions with Offenders Post-Assault and Their Impacts on Recovery: A Qualitative Study of Sexual Assault Survivors and Support Providers. J Aggress Maltreat Trauma.

[39] Sigurdardottir S, Halldorsdottir S (2021) Persistent Suffering: The Serious Consequences of Sexual Violence against Women and Girls, Their Search for Inner Healing and the Significance of the #MeToo Movement. Int J Environ Res Public Health 18(4) 10.3390/ijerph18041849 PMC7918207 33672865

[40] Smid GE, Lind J, Bonde JP (2022). Neurobiological mechanisms underlying delayed expression of posttraumatic stress disorder: A scoping review. World J Psychiatry.

[41] Soziales, B. f. A. u. (2023). „Soziale Entschädigung.“

[42] Sporer SL, Antonelli M (2022). Psychology of eyewitness testimony in Germany in the 20th century. Hist Psychol.

[43] Toglia MP, Schmuller J, Surprenant BG, Hooper KC, DeMeo NN, Wallace BL (2022). Novel Approaches and Cognitive Neuroscience Perspectives on False Memory and Deception. Front Psychol.

[44] Uzer T, Beşiroğlu L, Karakılıç M, Yalçın D, Yazar MS, İlden Koçkar A (2023). Investigating traumatic memory integration in people with and without post-traumatic stress disorder using the event-cueing paradigm. Memory.

[45] Vanderveren E, Bijttebier P, Hermans D (2017). The Importance of Memory Specificity and Memory Coherence for the Self: Linking Two Characteristics of Autobiographical Memory. Front Psychol.

[46] Visser RM, Lau-Zhu A, Henson RN, Holmes EA (2018) Multiple memory systems, multiple time points: how science can inform treatment to control the expression of unwanted emotional memories. Philos Trans R Soc Lond B Biol Sci 373(1742) 10.1098/rstb.2017.0209 PMC5790835 29352036

[47] Vissia EM, Lawrence AJ, Chalavi S, Giesen ME, Draijer N, Nijenhuis ERS, Aleman A, Veltman DJ, Reinders A (2022). Dissociative identity state-dependent working memory in dissociative identity disorder: a controlled functional magnetic resonance imaging study. BJPsych open.

[48] Volbert R, Schemmel J, Tamm A (2019) Die aussagepsychologische Begutachtung: eine verengte Perspektive? Forensische Psychiatr Psychol Kriminologie

[49] Williams SE, Ford JH, Kensinger EA (2022). The power of negative and positive episodic memories. Cogn Affect Behav Neurosci.

[50] Wolf A-K, Werner M (2021). Victims’ Rights Looking Good on Paper—How Criminal Prosecution in Germany Fails Victims of Sexual Violence. Ger Law J.

[51] Wu Y, Hartman D, Wang Y, Goldfarb D, Goodman GS (2023) Suppression and Memory for Childhood Traumatic Events: Trauma Symptoms and Non-Disclosure. Top Cogn Sci 10.1111/tops.12667 37352442

